# Orthodontic Management in Aggressive Periodontitis

**DOI:** 10.1155/2017/8098154

**Published:** 2017-02-16

**Authors:** Rajesh Gyawali, Bhagabat Bhattarai

**Affiliations:** ^1^Department of Orthodontics, College of Dental Surgery, B.P. Koirala Institute of Health Sciences, Dharan 56700, Nepal; ^2^Department of Periodontics, Kathmandu Medical College and Teaching Hospital, Kathmandu 44600, Nepal

## Abstract

Aggressive periodontitis is a type of periodontitis with early onset and rapid progression and mostly affecting young adults who occupy a large percentage of orthodontic patients. The role of the orthodontist is important in screening the disease, making a provisional diagnosis, and referring it to a periodontist for immediate treatment. The orthodontist should be aware of the disease not only before starting the appliance therapy, but also during and after the active mechanotherapy. The orthodontic treatment plan, biomechanics, and appliance system may need to be modified to deal with the teeth having reduced periodontal support. With proper force application and oral hygiene maintenance, orthodontic tooth movement is possible without any deleterious effect in the tooth with reduced bone support. With proper motivation and interdisciplinary approach, orthodontic treatment is possible in patients with controlled aggressive periodontitis.

## 1. Introduction

Periodontitis is the inflammation of the supporting tissues of the teeth, caused by specific microorganisms, which leads to progressive destruction of the periodontal ligament and alveolar bone with pocket formation, recession, or both. Aggressive periodontitis (juvenile or early onset periodontitis) is characterized by early onset mostly affecting young people and in the absence of significant plaque and calculus and shows a rapid progression of destruction. It has been found to show a familial pattern of occurrence with no known contributing medical history [1, 2].

Localized aggressive periodontitis is characterized by involvement of at least one first molar and one incisor or second molar and two or fewer canines or premolars with greater than 3 mm of attachment loss. Generalized aggressive periodontitis involves four or more teeth with greater than 3 mm of attachment loss; and at least two affected teeth were second molars, canines, or premolars [3].

Aggressive periodontitis affects adolescents and the percentage of adolescents is highest among orthodontic patients [4]. Hence, it is of utmost important for an orthodontist to have knowledge and ability to diagnose and manage the case of aggressive periodontitis with multidisciplinary approach.

## 2. Etiology and Epidemiology

Aggressive periodontitis shows its onset at puberty [5], may be because of the elevation of certain hormones in blood and gingival fluid, both of which may act as growth factors for the causative bacteria. Aggressive periodontitis is more common among younger individuals, but it does not mean that all youth are equally susceptible. Prevalence of aggressive periodontitis varies widely among various races and ethnicities from 0.1% to 15% [2, 6]. It is more prevalent in African Americans and Hispanics as compared to Caucasians [7–10]. Besides, genetics, age, and environment may also influence it. Females are found to be more affected than male [11].

The major pathogen found to be involved in aggressive periodontitis is* Aggregatibacter actinomycetemcomitans* (previously known as* Actinobacillus actinomycetemcomitans*). Besides other bacteria like* Porphyromonas gingivalis*,* Campylobacter rectus*,* Eikenella corrodens*, and* Capnocytophaga* sp. are also found to be associated with it. Even with these pathogens, the susceptibility of the disease differs among individuals and the reason behind is the immune defects.

Neutrophils are the major blood cells of innate defense active against bacterial pathogens and are derived from the bone marrow stem cells. In periodontium, these neutrophils are the protective cells and any defects associated with them lead to increased susceptibility to periodontitis. Neutrophil defects like abnormalities in adherence, chemotaxis, superoxide generation, phagocytosis, and bactericidal activities have also been found to be accountable for the susceptibility to aggressive periodontitis [2]. It is multifactorial in origin and is a genetically complex disease. It is found to be inherited by an autosomal dominant [12], autosomal recessive [13–15], and X-linked dominant mode [5, 16, 17].

## 3. Diagnosis of Aggressive Periodontitis

Diagnosis is based on history, clinical examination, and radiographs. Besides immunological tests, microbiological tests are also performed. More often, its diagnosis is made in the age which is the same age as that when orthodontic treatment is generally started [18]. The first molar which is usually involved in aggressive periodontitis is the tooth important for orthodontist because it is frequently used for anchorage in various removable or fixed appliance. Also contact of bands used in first molars with the gingival tissue may irritate it aggravating the condition [19]. Hence it is essential that orthodontist screens each case for aggressive periodontitis and is able to make an honest diagnosis and coordinate with the periodontist for proper care.

For the diagnosis of localized aggressive periodontitis, there should be attachment loss of at least 4 mm on at least one permanent first molar and one incisor [20]. Here are some features which help in the diagnosis of aggressive periodontitis.

### 3.1. Clinical [21]

- Generally affecting the young adults.
- Rapid loss of attachment and alveolar bone height.
- Familial aggregation.
- Absence of significant medical history.
- Severe periodontal destruction which is inconsistent with the amount of deposits.
- Lack of clinical signs of inflammation in spite of the presence of deep pockets and bone loss.

### 3.2. Radiographic [3]

- Vertical loss of alveolar bone height around first molar and incisors.
- Arc shaped bone loss seen extending from the distal surface of the second premolar to the mesial surface of the second molar.

### 3.3. Microbiologic [22]

Although a large number of pathogens are associated with periodontitis, many studies have shown few specific strains related to aggressive periodontitis.* A. actinomycetemcomitans* is found in high frequency in microbial deposits on the teeth affected with localized aggressive periodontitis. Similarly, patients with generalized aggressive periodontitis show high subgingival levels of* Selenomonas* sp. and* T. lecithinolyticum*.

### 3.4. Host Susceptibility [2]

Some people are more susceptible to aggressive periodontitis indicating a significant role of host susceptibility behind the disease. In addition, local and general environmental factors also play significant roles. Herpes virus may increase the host susceptibility and aggravate the severity of aggressive periodontitis.

## 4. Orthodontic Problems Associated with the Aggressive Periodontitis

The loss of interproximal attachment is the main factor leading to pathologic tooth migration [23] and clinically seen as extrusion, proclination, rotation, diastema, or drifting into edentulous space [24]. Altered level of the incisal edge and space between anterior teeth leads to unaesthetic appearance and is the prime reason for seeking orthodontic treatment.

## 5. Role of Orthodontist in the Management of Aggressive Periodontitis

Aggressive periodontitis may develop before, during, or even after the completion of orthodontic treatment. So periodontal screening should be done in all patients regardless of the initiation of orthodontic treatment [25]. Khocht et al. found that periodontal screening and recording (PSR) is an effective tool to scrutinize periodontal diseases [26].

### 5.1. Before Starting Orthodontic Treatment

For any patient attending the orthodontic clinic, the orthodontists should ensure that their oral hygiene is good. In patients with poor oral hygiene, oral prophylaxis is recommended and their ability to maintain the hygiene is evaluated. If the compliance towards the oral hygiene is poor, orthodontic therapy is postponed till the achievement of proper plaque control.

Mathews and Kokich recommended that, during clinical examination, at least five minutes of periodontal examination, including probing of key indicator teeth, is mandatory before making orthodontic diagnosis and treatment planning [27]. Gingival inflammation, swelling, recession, tooth mobility, and pathological pockets should be assessed. Besides, the patient should be asked for the recent change in the position or drifting of teeth. Radiographic assessment of bone loss is made from the bite wing radiograph of the first molar and intraoral periapical radiograph of incisors [19]. Newer imaging methods like CBCT give more insight into the bony defects which facilitates better treatment planning [28]. Further, serum IgG titer test against each pathogenic bacterium can be done with ELISA as the serum level of IgG directly correlates with the number of pathogenic bacteria in the periodontium [29]. Elimination of bacteria from the periodontal pockets can be assessed with this test which is useful for determining the optimal timing to start orthodontic treatment [30]. Any clinical signs of aggressive periodontal disease like attachment loss, pockets, and bone loss when found should be consulted with a periodontist for appropriate care.

After the patient is diagnosed with aggressive periodontitis, he/she should be referred to a periodontist. Along with the local mechanical debridement, adjunctive antibiotics are a valuable tool in the management; however, the literature is not very clear regarding optimum dosages, duration, and timing [31]. An adjunctive course of seven days of amoxicillin (500 mg) and metronidazole (500 mg) three times a day can significantly improve clinical outcome of aggressive periodontitis treated nonsurgically [32]. Flap surgeries for debridement either with resective or regenerative procedures can be performed to get assess for instrumentation [33].

A follow-up period of 6 months is needed after active periodontal therapy so as to observe the resolution of active inflammation and restoration of the periodontal health [34, 35]. As there is no deleterious effect of orthodontic tooth movement on healthy periodontium, orthodontic treatment can be started once the health of periodontium is achieved back [36]. Now it is the duty of the orthodontist to motivate the patient to maintain good oral hygiene during the course of orthodontic treatment [37] and warn the patient regarding the consequences of poor oral health.

The decrease in the height of the alveolar bone around the affected teeth demands change in orthodontic treatment plan and biomechanics. As the bone surrounding the root surface is decreased, the periodontal ligament area also gets decreased. Hence, even less force applied may produce greater pressure in the periodontal ligament and thus increases the extrusive component of the applied force. Hence, light force should be used to move the tooth with reduced bone support. As the centre of resistance is shifted apically, moment of the applied force increases and thus large moments are needed to control the root movement [38].

Fixed orthodontic appliance is preferred over removable because fixed appliance acts as splinting, helps in stabilizing anchorage, and provides light continuous force which is not possible with removable appliances. Also, applying moments to control root movements is easier with fixed appliance.

Most of the removable and fixed orthodontic appliances use first molar as an anchorage unit. Reduced bone support of the first permanent molar in aggressive periodontitis decreases its anchorage quality leading to undesirable side effects. If anchorage is critical, temporary anchorage devices (TAD) can be used in such cases. Also direct application of force from TAD avoids bonding/banding of anchorage segments reducing plaque retentive areas [39]. However, it is very important to motivate patient to maintain proper oral hygiene as oral inflammation leads to failure of temporary anchorage devices [40, 41].

### 5.2. During Active Orthodontic Treatment

Orthodontic treatment should be started only after the clinician is convinced that the patient is well motivated and can follow the oral hygiene instructions well. Orthodontic therapy includes wires, brackets, bands, or other several components which further lodge plaque and make oral hygiene more challenging. Hence, the importance of rigorous oral hygiene should be reinforced in each visit of the patient. Orthodontic patients with aggressive periodontitis require a separate periodontal appointment with a periodontist once every 3 months [19].

However, clinical periodontal examination should be done in every appointment patient comes for orthodontic follow-up. Any detrimental effects in a patient are noted and if found severe the patient is referred to a periodontist immediately. For any patient who cannot maintain proper oral hygiene, active orthodontic force should be stopped and removal of the whole appliance may be considered till the desired improvement and motivation are achieved. The orthodontic treatment may be continued once the condition is improved. Radiographic examination, including bitewing for the molars and the intraoral periapical radiograph for the incisors, should be taken once a year during the course of orthodontic treatment [19].

Sometimes aggressive periodontitis can be diagnosed during the course of orthodontic treatment. In such condition, the active orthodontic force should be discontinued immediately and the patient should be referred to a periodontist for proper therapy [42]. To facilitate the proper periodontal therapy, removal of the orthodontic appliance should be considered if necessary [36].

The orthodontic treatment should be performed with great care in patients with aggressive periodontitis. Orthodontic bands predispose to gingivitis, gingival enlargement, increased pocket depth, and even attachment loss due to mechanical irritation or poor oral hygiene [43, 44]. “Special periodontally friendly bands” are being designed which is more hygienic than the conventional bands [45]. Bondable Buccal tubes are preferred over bands because they have minimal negative effects on the periodontium as they are at a greater distance from the gingival margins [46, 47]. Whenever possible, steel ligature wire should be used instead of elastomeric modules to ligate the archwire to the brackets because the former is less plaque retentive and easier to clean [48–50]. Special consideration should be given to remove excess composite around the brackets especially at the gingival margin.

Placing brackets in an ideal position in all teeth may not be necessary, particularly in the anchorage segment involving teeth with bone loss. Brackets are bonded in such a way that the slots are aligned and wire can be engaged passively so as to minimally disturb the anchor teeth in physiologically satisfactory position [35]. Treatment should be done with the use of simplest system avoiding multiple wires, loops, and multiple attachments to allow better oral hygiene [51]. During the stage of levelling and alignment, light wires like 0.012′′ or 0.014′′ Nickel titanium are recommended [18]. Activation of orthodontic appliance in patient with aggressive periodontitis should be done at longer interval as the periodontal remodeling takes more time when compared to normal individual.

If orthodontic plan includes extraction, then the teeth with hopeless prognosis are extracted for which change in the original mechanotherapy may be needed. After extraction of teeth involved in aggressive periodontitis, it is better to wait at least 3 months to move a sound tooth in the extraction space to prevent reinvolvement of newly positioned tooth [52].

Teeth bonded with self-ligating brackets had fewer bacteria in plaque as compared with elastomeric rings [53, 54]. However, brackets ligated with ligature wire harbor still less bacteria than the self-ligating brackets which may be because of the clips and retentive areas in self-ligating brackets [54]. Hence, metallic ligature wire seems to be appropriate in cases of patients with aggressive periodontitis.

There is difficulty in maintaining oral hygiene with lingual appliance as compared to labial [55–57].* A. actinomycetemcomitans* is associated with aggressive periodontitis, which is found in very high frequency among orthodontic patients [58, 59]. As oral hygiene is critical during management of patients with aggressive periodontitis, labial appliance can be preferred. However, increased prevalence of* A. actinomycetemcomitans* and* P. gingivalis* has not been observed in patients with lingual appliance [60].

The orthodontist may need to intrude the extruded incisor with vertical bone loss involved in the aggressive disease. Improvement in alveolar bone defects, probing depth, gingival esthetics, and clinical attachment gain can be seen with proper application of orthodontic force [36, 61]. To prevent the apical displacement of plaque, therapy aimed to reduce pocket depth should be done earlier [62].

### 5.3. During Retention and Long Term Follow-Up

Removable retainers are best if periodontal health is considered, but poor compliance may result in relapse. Also, clear vacuum formed retainer which has minimal gingival contact would be better than the acrylic removable retainer with retentive clasps in close proximity with the gingiva. Fixed retainers placed after active orthodontic treatment may contribute to plaque retention [63]. So, periodontal examination should be done as a part of follow-up. Those patients who have not developed aggressive periodontitis till the end of active orthodontic therapy may develop during the retention phase, so similar precautions should be taken in all the patients. Regular periodontal follow-up along with strict oral hygiene measures contribute to long term maintenance of the achieved result [64, 65]. Successful orthodontic therapy can be done despite the compromised periodontium with aggressive periodontitis (Table 1).

## 6. Conclusion

Aggressive periodontitis mostly affects young adults, causing rapid periodontal destruction with loss of supporting alveolar bone. For the movement of teeth, healthy periodontium is required. Hence, in patients with aggressive periodontitis, orthodontic treatment is possible only when the disease is brought under control via careful monitoring before, during, and after the active therapy.

## Figures and Tables

**Table 1:** Summary of the case reports in the orthodontic management of aggressive periodontitis.

Author (year)	Patient		Aggressive periodontitis	Type of periodontal procedures done	Duration of active orthodontic treatment	Orthodontic considerations during treatment
	Sex	Age				
Ishihara et al. [30] (2015)	F	21	Generalized	Surgical and nonsurgical	28 months	(i) Close monitoring of serum IgG against *A. actinomycetemcomitans* and *Prevotella intermedia* (ii) Preadjusted edgewise appliance with self-ligating brackets (iii) Initial levelling with 0.012 nickel titanium wire (iv) Lingual bonded retainer as well as wrap around retainer after active therapy

Closs et al. [65] (2010)	F	22	Localized	Surgical and nonsurgical	32 months	(i) Extraction of maxillary deciduous second molars followed by mesial movement of maxillary molars (missing maxillary second premolars) (ii) Segmented mechanics and light force (iii) Removable retainer in maxillary while fixed lingual retainer in mandibular arch

Miyamoto et al. [66] (2008)	F	24	Localized	Nonsurgical	40 months	(i) Extraction of maxillary first premolars and subapical osteotomy to reposition it back (ii) Extraction of mandibular central incisor and proximal stripping of other incisors to minimize tooth movement in lower arch (iii) Flexible spiral wire and removable circumferential retainer

Passanezi et al. [67] (2007)	F	17	Localized	Surgical and nonsurgical	26 months	(i) Initial levelling with light force (0.012 NITI) (ii) Intrusion of flared and extruded maxillary incisors with reverse curve wire (iii) Class II elastics for overjet control (iv) Permanent fixed retention in both arches with removable retainer in maxillary arch

Craddock et al. [68] (2007)	F	27	Generalized	—	7 months	(i) Alignment and levelling of the drifted upper incisors with fixed appliance (ii) permanent bonded retainer

Ogino et al. [69] (2006)	F	30	Localized	Surgical and nonsurgical	19 months	(i) Intrusion of maxillary and mandibular incisors to reduce overbite (ii) Retraction of maxillary incisors to reduce overjet (iii) Begg retainer in maxillary arch and fixed retainer in mandibular arch

Maeda et al. [70] (2005)	F	27	Localized	Surgical and nonsurgical	21 months	(i) Extraction of 11 and 31 (ii) Levelling and alignment started with 0.012 Nickel titanium archwire (iii) Cervical pull headgear (iv) Multiloop edgewise archwire (v) Maxillary circumferential removable retainer and mandibular bonded canine to canine retainer

Okada et al. [71] (2000)	M	17	Localized	Nonsurgical	36 months	(i) Patient presented with Pierre robins sequence (ii) Extraction of maxillary first premolars and retraction and alignment of upper anteriors (iii) During levelling, maxillary left central incisor showed marked mobility and thus is extracted (later replaced with fixed bridge). (iv) Active orthodontic therapy was halted for half a year (v) Simultaneous use of quad helix for maxillary arch expansion (vi) Wrap around retainer

Harpenau and Boyd [64] (2000)	F	16	Localized	Nonsurgical	—	(i) Extraction of all first molars and subsequent space closure with retraction and well as protraction

Folio et al. [72] (1985)	M	32	Localized	—	4 months	(i) Supra-erupted maxillary left lateral incisor (ii) 0.019 by 0.022 inch passive arch wire with 0.018 inch round wire “kicker” for intrusive force (iii) Mild root resorption of the intruded tooth maintaining the vitality (iv) Upper Hawley's appliance for retention

Folio et al. [72] (1985)	M	21	Localized	—	4 months	(i) Maxillary left lateral incisor was in crossbite which was corrected with 0.016′′ Nickel titanium wire (ii) Modified Hawley's retainer after alignment

Folio et al. [72] (1985)	F	28	Localized	—	11 months	(i) Extensively drifted maxillary incisors were aligned and space closure by 0.018 by 0.022 inch wire with closing loops

Folio et al. [72] (1985)	F	16	Localized	—	19 months	(i) Intrusion of mandibular incisors and alignment of maxillary anteriors (which was pathologically migrated) (ii) Removable Hawley's retainer

McLain et al. [73] (1983)	F	12	Localized	Nonsurgical	36 months	(i) Extraction of severely affected teeth (all first molars and four lower incisors and left maxillary central incisor) (ii) Mesialization of second and third molars 3 yrs after the extraction (iii) Upper 17 × 22 and lower 17 × 25 wire with loops were used to close the space
